# Integrating oculomics with genomics reveals imaging biomarkers for preventive and personalized prediction of arterial aneurysms

**DOI:** 10.1007/s13167-023-00315-7

**Published:** 2023-02-13

**Authors:** Yu Huang, Cong Li, Danli Shi, Huan Wang, Xianwen Shang, Wei Wang, Xueli Zhang, Xiayin Zhang, Yijun Hu, Shulin Tang, Shunming Liu, Songyuan Luo, Ke Zhao, Ify R. Mordi, Alex S. F. Doney, Xiaohong Yang, Honghua Yu, Xin Li, Mingguang He

**Affiliations:** 1 Guangdong Eye Institute, Department of Ophthalmology, Guangdong Provincial People’s Hospital (Guangdong Academy of Medical Sciences), Southern Medical University, Guangzhou, 510080 China; 2 Guangdong Cardiovascular Institute, Guangdong Provincial People’s Hospital (Guangdong Academy of Medical Sciences), Southern Medical University, Guangzhou, 510080 China; 3 Division of Population Health and Genomics, University of Dundee, Ninewells Hospital and Medical School, Dundee, DD1 9SY UK; 4 State Key Laboratory of Ophthalmology, Zhongshan Ophthalmic Center, Sun Yat-sen University, Guangzhou, 510060 China; 5 Centre for Eye Research Australia, Melbourne, VIC 3002 Australia; 6 Department of Radiology, Guangdong Provincial People’s Hospital (Guangdong Academy of Medical Sciences), Southern Medical University, Guangzhou, 510080 China; 7 Guangdong Provincial Key Laboratory of Artificial Intelligence in Medical Image Analysis and Application, Guangdong Provincial People’s Hospital (Guangdong Academy of Medical Sciences), Southern Medical University, Guangzhou, 510080 China; 8 Division of Molecular and Clinical Medicine, School of Medicine, University of Dundee, Dundee, Ninewells Hospital and Medical School, Dundee, DD1 9SY UK; 9 Department of Emergency Medicine, Guangdong Provincial People’s Hospital (Guangdong Academy of Medical Sciences), Southern Medical University, Guangzhou, 510080 China

**Keywords:** Predictive preventive and personalized medicine (PPPM / 3PM), Aneurysm, Oculomics, Retinal vascular features, Phenome-wide association analysis, Genetic risk scores, Risk assessment, Imaging biomarker

## Abstract

**Objective:**

Arterial aneurysms are life-threatening but usually asymptomatic before requiring hospitalization. Oculomics of retinal vascular features (RVFs) extracted from retinal fundus images can reflect systemic vascular properties and therefore were hypothesized to provide valuable information on detecting the risk of aneurysms. By integrating oculomics with genomics, this study aimed to (i) identify predictive RVFs as imaging biomarkers for aneurysms and (ii) evaluate the value of these RVFs in supporting early detection of aneurysms in the context of predictive, preventive and personalized medicine (PPPM).

**Methods:**

This study involved 51,597 UK Biobank participants who had retinal images available to extract oculomics of RVFs. Phenome-wide association analyses (PheWASs) were conducted to identify RVFs associated with the genetic risks of the main types of aneurysms, including abdominal aortic aneurysm (AAA), thoracic aneurysm (TAA), intracranial aneurysm (ICA) and Marfan syndrome (MFS). An aneurysm-RVF model was then developed to predict future aneurysms. The performance of the model was assessed in both derivation and validation cohorts and was compared with other models employing clinical risk factors. An RVF risk score was derived from our aneurysm-RVF model to identify patients with an increased risk of aneurysms.

**Results:**

PheWAS identified a total of 32 RVFs that were significantly associated with the genetic risks of aneurysms. Of these, the number of vessels in the optic disc (‘ntreeA’) was associated with both AAA (*β* = −0.36, *P* = 6.75e−10) and ICA (*β* = −0.11, *P* = 5.51e−06). In addition, the mean angles between each artery branch (‘curveangle_mean_a’) were commonly associated with 4 MFS genes (*FBN1*: *β* = −0.10, *P* = 1.63e−12; *COL16A1*: *β* = −0.07, *P* = 3.14e−09; *LOC105373592*: *β* = −0.06, *P* = 1.89e−05; *C8orf81/LOC441376*: *β* = 0.07, *P* = 1.02e−05). The developed aneurysm-RVF model showed good discrimination ability in predicting the risks of aneurysms. In the derivation cohort, the *C*-index of the aneurysm-RVF model was 0.809 [95% CI: 0.780–0.838], which was similar to the clinical risk model (0.806 [0.778–0.834]) but higher than the baseline model (0.739 [0.733–0.746]). Similar performance was observed in the validation cohort, with a *C*-index of 0.798 (0.727–0.869) for the aneurysm-RVF model, 0.795 (0.718–0.871) for the clinical risk model and 0.719 (0.620–0.816) for the baseline model. An aneurysm risk score was derived from the aneurysm-RVF model for each study participant. The individuals in the upper tertile of the aneurysm risk score had a significantly higher risk of aneurysm compared to those in the lower tertile (hazard ratio = 17.8 [6.5–48.8], *P* = 1.02e−05).

**Conclusion:**

We identified a significant association between certain RVFs and the risk of aneurysms and revealed the impressive capability of using RVFs to predict the future risk of aneurysms by a PPPM approach. Our finds have great potential to support not only the predictive diagnosis of aneurysms but also a preventive and more personalized screening plan which may benefit both patients and the healthcare system.

**Graphical abstract:**

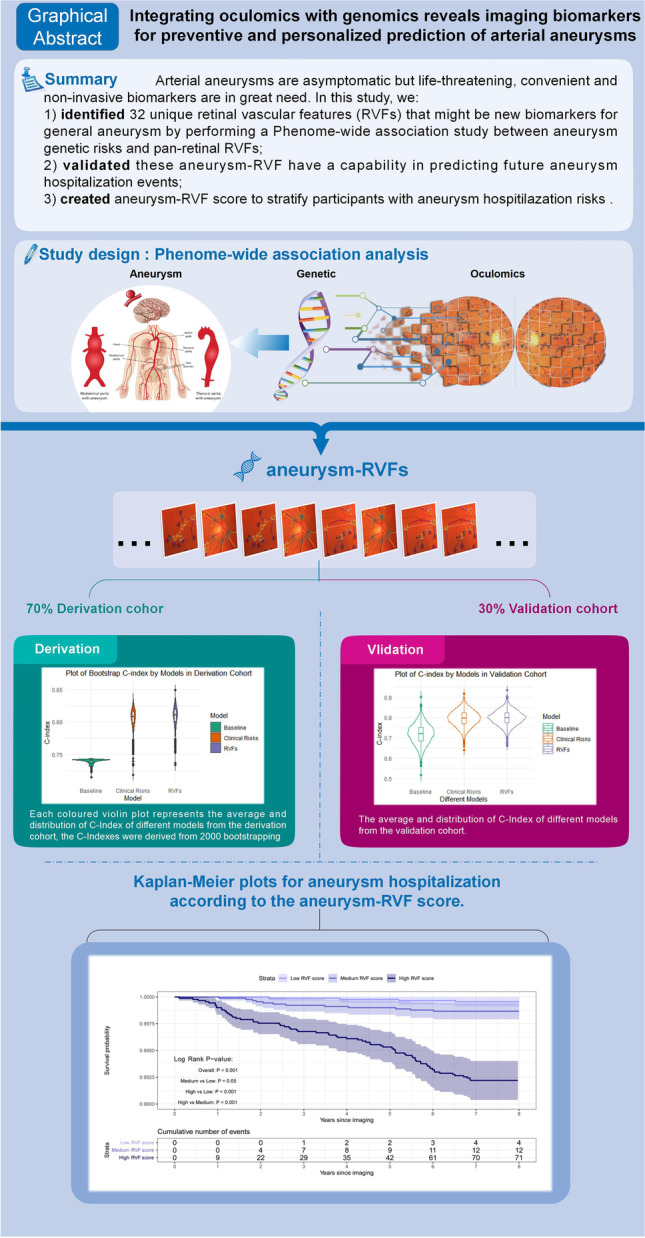

**Supplementary Information:**

The online version contains supplementary material available at 10.1007/s13167-023-00315-7.

## Introduction

### Biomarkers of arterial aneurysm are needed in the realm of prediction, prevention and personalized medicine (PPPM)

Arterial aneurysm is one of the most common diseases affecting the artery after atherosclerosis and represents a severe condition due to the increased risk of dissection or rupture which has a mortality rate greater than 80% [1, 2]. According to data reported by the ‘2019 Global Burden of Disease’ study, the global number of aneurysm-related deaths is expected to increase by 42% to achieve 244,685 in 2030, compared to 172,426 in 2019 [3]. Irrespective of severity, the onset of aneurysms is usually asymptomatic. Therefore, current medical care strategies for aneurysms need to be improved, especially in terms of predictive diagnosis and targeted screening, such improvement may permit early prevention and personalized management [4].

Effective and safe biomarkers are needed for predicting aneurysms or the development of preventive screening protocols. According to current international guidelines, only a limited number of general biomarkers, such as age and sex, have been applied to facilitate routine aneurysm screening [5, 6]. However, these biomarkers neglect the heterogeneous characteristics of the disease [7–11] and are not precise enough to benefit individuals who do not meet the current screening criteria. A recent study proposed an effective and accurate screening approach for abdominal aortic aneurysm (AAA) based on a broader spectrum of clinical biomarkers [12], thereby providing a foundation for developing and testing new biomarker-based screening approaches for aneurysm. Therefore, the integration of numerous biological data, such as genetics and medical images, and the identification of non-invasive and convenient biomarkers, could facilitate the prediction of aneurysm and provide options for personalized medical prevention strategies, which is an essential step in the context of PPPM [4].

### Genomics is a fundamental consideration for PPPM

Exploring biomarkers associated with aneurysms identified from routinely collected electronic health records might suffer from misclassification bias due to the asymptomatic nature of the disease. Genomics can provide unique data about an individual’s unique risk of disease [13, 14] and genetic risks of aneurysms were stable and precise, which might assist in identifying robust biomarkers [15, 16]. There have been several large-scale genome-wide association studies (GWAS) that report a potential shared genetic aetiology among the main types of aneurysms [17–21], including AAA, thoracic aortic aneurysm (TAA), intracranial aneurysm (ICA) and Marfan syndrome (MFS) in which the main complication is TAA [22, 23]. Therefore, the genetic risk of aneurysm can be a reasonably precise proxy for aneurysms since this risk can (i) better reflect the biological mechanism of aneurysms and (ii) better represent an individual’s risk and reduce misclassification bias due to the delay or lack of aneurysm detection.

### Oculomics features imaging biomarkers of aneurysms and is an emerging tool for PPPM

Identifying medical imaging biomarkers is a key component in PPPM since they are essential to patient-tailored disease prediction or therapy [24]. For aneurysm screening or prediction, the use of image biomarkers derived from ultrasound or computer tomography (CT) is not cost-effective and can expose patients to radioactive agents, thus limiting the broad application of these techniques as routine healthcare processes. Oculomics is an emerging research area that utilizes ocular information derived from non-invasive and easily accessible ocular examinations to gain insights into systemic health. Some researchers have reported the use of oculomics in the context of PPPM [25], especially for the use of oculomics of retinal vascular morphological features (RVFs) as prediction biomarkers for cardiovascular diseases (CVDs) [26–29]. However, only a few studies have investigated their relationship with aneurysms [30, 31]. Since aneurysms mainly demonstrate irreversible changes in the vascular morphology, we hypothesized that the oculomics of RVF may contains more imaging biomarkers for aneurysm than for other CVDs and these RVFs may facilitate the early identification of patients with a high risk of aneurysm from the perspectives of PPPM. This strategy will benefit vulnerable populations, especially those that find it difficult to reach advanced medical resources [32].

### Working hypothesis

Compared to clinical phenotypic data, oculomics derived from retinal fundus image is considered more convenient and efficient, non-invasive, with lower costs (for all resources), and more importantly, reflects systemic vascular properties. Previously, we developed a deep learning algorithm, the Retinal-based Microvascular Health Assessment System (RMHAS) [33] that can generate data on the omics scale from RVFs, thus providing more comprehensive oculomics of RVFs. In this study, personalized genetic risk of aneurysm was integrated with the oculomics by phenome-wide association analysis (PheWAS) to identify biological meaningful aneurysm-RVF biomarkers. Followed by testing their capability in differentiating aneurysms, this study aimed to investigate the value of oculomics in (i) providing imaging biomarkers for the predictive diagnosis of arterial aneurysms and (ii) identifying subjects at high risk and supporting early detection of arterial aneurysms. This could be particularly useful as it might allow us to develop a more refined and targeted screening approach for aneurysm that aligns with the aims of PPPM.

## Methods

### Study population

The UK Biobank is a large-scale and prospective cohort study with over 500,000 participants aged 40–69 years that were recruited between 2006 and 2010. This study collected extensive phenotypic and genotypic data from each participant with their informed consent. Further details of the UK Biobank data and the protocols involved have been described elsewhere [34]. In brief, a total of 502,505 individuals in 22 assessment centres across the UK agreed to participate in this study (a response rate of 5.5%). During baseline assessment, participants completed comprehensive questionnaires, provided a range of physical measurements and provided biological samples. Detailed health-related events were achieved through linkage to national electronic health record datasets. Ophthalmic assessments, including retinal fundus photography, were introduced to the baseline assessment in 2009 for six assessment centres. Participants without fundus imaging or genetic data were excluded.

This study was approved by the National Information Governance Board for Health and Social Care and the NHS Northwest Multicentre Research Ethics Committee (Reference: 11/NW/0382) and the Biobank consortium (Application number: 62489). Since de-identified data in a public dataset was used, the Medical Research Ethics Committee of Guangdong Provincial People’s Hospital waived the requirements to obtain ethical approval.

### Genetic risk of aneurysm

In total, 488,377 participants were genotyped by the UK Biobank Axiom genotyping array. Stringent quality control was performed and genotype imputation was carried out using the Haplotype Reference Consortium (HRC) reference panel; further details relating to the genotype and quality control information were described by Bycroft et al. [35].

Genetic information for abdominal, thoracic and intracranial aneurysms, and MFS, were taken from genome-wide association studies (GWAS) for the corresponding traits. For AAA, 12 genetic loci associated with AAA were identified from a meta-analysis of 4972 cases and 99,858 controls [17]; for TAA, we used three single nucleotide polymorphisms (SNPs) that were found to be associated with TAA in a previous GWAS of 1351 affected individuals and 18,295 controls [19]; and for intracranial aneurysms, we used 17 SNPs that were previously identified by a GWAS of 10,754 cases and 306,882 controls [18]. These SNPs were used to generate different weighted genetic risk scores (GRSs) for each participant using the --score function implemented in PLINK 2.0 [36]. The magnitude of the association with different aneurysms (GWAS beta coefficient) was used as the weighting factor for each variant included in the GRS.

In the analysis of MFS, we selected five MFS SNPs that were allocated to different genes [20, 21]. rs10519177 was located in the region of *FBN1*, a common MFS gene, while the other four SNPs rs2297676 (located within *COL16A1*), rs1432302 (located within *LOC105373592*), rs3020167 (located between *C8orf81* and *LOC441376*) and rs2278601 (located within *SMAD6*) were considered to be associated with the extreme arterial phenotype of MFS such as thoracic aneurysm or dissection. The genotypes of these SNPs were extracted by PLINK 2.0 for each participant using the --extract and --recode functions.

### Oculomics of retinal vascular features

The 45-degree non-mydriatic retinal fundus and optical coherence tomography (OCT) images of the optic disc and macular were captured using a spectral domain OCT (Topcon 3D OCT 1000 Mk2, Topcon Corp, Tokyo, Japan) for each eye. A total of 131,238 fundus images were obtained from 66,500 participants. Only the retinal fundus images from the right eye were used for analysis. A machine learning system, referred to as the Retinal-based Microvascular Health Assessment System (RMHAS), was previously developed and validated to automatically and quickly extract and quantify retinal microvascular features [33]. For each image, pan-retinal vessel geometric parameters, such as calibre, complexity, length, tortuosity and branching angle were quantified. A demonstration of the algorithm is publicly available at: https://www.retinavessel.com/ (Supplementary Method 1).

Compared to previous retinal vessel analysis tools, RMHAS can extract pan-retinal vessel features and generate 91 RVFs for each fundus image. Details of the analysing pipeline and the 91 RVFs are given in Supplementary Figure 1 and Supplementary Table 1.

### Characteristics associated with the risk of aneurysm

According to previous prediction models for aneurysm [12, 37], modifiable clinical risk factors, demographic information and social economic status were considered as characteristics that can be used to predict aneurysm events. Baseline characters included age, sex, systolic (SBP) and diastolic (DBP) blood pressure, blood lipid, glycated haemoglobin, smoking status, body mass index (BMI), baseline cardiovascular disease (defined by International Classification of Diseases [ICD-10] codes I20–I25 and I60–I69 excluding I67.0 and I67.1), baseline diabetes (self-reported type 1 or type 2 diabetes), self-reported hyperlipidaemia and the use of blood pressure- or cholesterol-lowering medications or anti-diabetic medications. Refractive error was measured using a Tomey RC 5000 autorefractor and data from the right eye was used for analysis (Supplementary Method 2 and Supplementary Table 2).

### Identifying aneurysm-RVF associations by PheWAS

PheWAS was performed between RVFs and each aneurysm GRS or SNP. In general, each RVF was regressed against a GRS or a SNP while adjusting for age and sex in the main analysis and additionally adjusted for SBP, Townsend deprivation score, smoking and refractive error in the sensitivity analysis. To adjust for multiple testing (we run 91 regressions for each PheWAS), Bonferroni correction was applied thus a *P* value less than 5.50e−04 (0.05/91) was considered statistically significant. The R package ‘PheWAS’ [38] was used to perform statistical analysis. Venn diagrams were used to identify the overall and shared aneurysm-RVFs driven by the effect of aneurysm or arterial dissection genes.

### Capability of aneurysm-RVF associations in predicting aneurysm risk

#### Assessment of aneurysm risk

The outcome was an 8-year risk of hospital admission due to aneurysm or symptomatic aneurysm defined by the earliest recorded event of fatal or non-fatal aneurysm since recruitment. Subjects were selected by their ICD10 code, OPCS4 (Classification of Interventions and Procedures) code, death record and self-report disease information. From the UK Biobank data set, data field ‘41270’, ‘40001’ and ‘40002’ were selected to define participants as ‘aneurysm dissection’, ‘thoracic aneurysm’, ‘abdominal aneurysm’, ‘thoracoabdominal aneurysm’, ‘brain aneurysm’ or ‘other aneurysm’ by ICD-10 code ‘I71-I72’, ‘I671’ or ‘I670’. Data field ‘41272’, ‘41200’ and ‘41210’ were also selected to define participants who had aneurysm surgery by OPCS4 code defined as ‘L18’, ‘L19’, ‘L27’, ‘L28’, ‘L424’, ‘L464’, ‘L254’, ‘L33’, ‘L48’, ‘L49’, ‘L533’, ‘L56’, ‘L57’, ‘L623’, ‘L624’ and ‘L705’. Finally, data fields ‘20002’ and ‘20004’ were selected to define self-reported aneurysm by choosing codes ‘1425’, ‘1492’ and ‘1591’ and ‘1592’ (Supplementary Table 3).

To derive the survival model, the recruitment date served as the beginning of the time at risk for each participant, and the period at risk terminated on the earlier of the first qualifying aneurysm event or the end of follow-up. The retinal images were taken between 2009 and 2010, and the disease records ended in 2018. Hence, follow-up was limited to a maximum of 8 years for each participant for the 8-year aneurysm risk.

#### Model derivation and validation

At first, participants with missing clinical risk data or RVFs were excluded; this resulted in 26,964 participants with a complete dataset in the cohort. Then, the complete cohort was divided into a derivation cohort (70% of the cohort, *n* = 18,954) and a validation cohort (30% of the cohort, *n* = 8010). We developed three models, one was the ‘baseline model’, including baseline age and sex as covariates; and the other two models with two distinct covariate sets, one including an additional 20 clinical risk factors, the other with the aneurysm-RVFs identified by previous PheWAS (Supplementary Method 3). We constructed a Cox model for these three models in the derivation cohort, and then run these separately in the validation cohort. The performance of the Cox models, over 8 years of follow-up, was tested by Harrell’s *C*-index separately in both the derivation and validation cohorts, using 2000 bootstraps (performed by the R package ‘rms’). The 95% CIs were also calculated based on the bootstrapping runs.

#### The predictive capability of aneurysm-RVF score

Finally, based on the metric of the aneurysm-RVF model in the derivation cohort, the survival probability was calculated for each participant in the complete cohort and used as an aneurysm-RVF score. This score was categorized into tertiles and the difference in the time-to-aneurysm probability across each tertile of the score was evaluated using Kaplan-Meier curve; log-rank tests were used to calculate *P* values. For sensitivity analysis, participants who have ever been in hospital due to aneurysm were removed, and the association of the aneurysm-RVF score with the first aneurysm was assessed.

### Statistical analysis

Continuous variables are presented as mean and standard deviation (SD) if approximately symmetrically distributed, and median and interquartile range (IQR) if skewed. Categorical variables are presented as counts and percentages. All analyses were two-sided and a *P* value of <0.05 was considered statistically significant. All analyses were performed using R 4.0.4 software or Stata14.

## Results

### Baseline population

The baseline cohort contained 51,597 participants with retinal vascular measurements and genetic information; these data were analysed by PheWAS. At the time the retinal images were taken, the median age of the participants was 56.0 (IQR: 14) years, 54.9% were female and 86.2% were white. With regard to clinical measurements, the median SBP was 135 (IQR: 24.5) mmHg, and DBP was 81.5 (IQR: 14). Median total cholesterol was 5.65 (IQR: 1.49) mmol/L, while for HDL, LDL and triglycerides, the mean values were 1.43 (IQR: 0.512) mmol/L, 3.50 (IQR: 1.15) mmol/L and 1.41 (IQR: 1.02) mmol/L, respectively. Mean HbA1C was 35.1 (IQR: 5.1) mmol/mol. The population had a relatively elevated BMI (26.6 [IQR: 5.71] kg/m^2^) and 56.3% had never been a smoker. Regarding medication history, 20.3% had never taken blood pressure-lowering drugs, 3.5% had taken blood glucose-lowering medication and 16.9% had taken lipid-lowering medication. On average, 2244 (4.3%) of the participants had a history of CVD, 548 (1.1%) reported a history of aneurysm, 374,485 (72.6%) had been diagnosed with hypertension, 2505 (4.8%) had diabetes and 23,145 (44.9%) had hyperlipidaemia. The median refractive status was 0.05 (IQR: 2.23) dioptre. Out of the total number of participants, 201 had an aneurysm event after recruitment (Table 1).

**Table 1:** Baseline characteristics of the participants

Continuous variable	*N* (all)	Median	IQR
Age (years)	51,597	57	14
BMI (kg/m^2^)	51,360	26.6	5.71
Townsend deprivation	51,527	−1.68	4.33
SBP (mmHg)	51,422	135	24
DBP (mmHg)	51,422	81.5	14
HbA1c (mmol/mol)	47,810	35.1	5.1
Total cholesterol (mmol/L)	48,219	5.65	1.49
HDL (mmol/L)	45,980	1.43	0.51
LDL (mmol/L)	48,138	3.50	1.15
Triglycerides (mmol/L)	48,173	1.41	1.02
Refractive error (D)	51,096	0.05	2.23
Category variable	*N* (all)	*N*	Percentage
Sex	51,597		
Female		28,303	54.90%
Male		23,294	45.10%
Ethnicity	51,484		
Non-white		7118	13.80%
White		44,366	86.20%
Smoker	51,294		
Never		29,064	56.3%
Former		17,434	33.8%
Current		4796	9.3%
Blood pressure lowing drugs (yes)	51,597	10,463	20.3%
Blood glucose lowing drugs (yes)	51,597	1826	3.5%
Blood lipid lowing drugs (yes)	51,597	8742	16.9%
Previous history of CVD (yes)	51,597	2244	4.3%
Previous history of aneurysm (yes)	51,597	548	1.1%
Previous history of hyperlipidaemia (yes)	51,597	23,145	44.9%
Previous history of hypertension (yes)	51,597	37,485	72.6%
Previous history of diabetes (yes)	51,597	2502	4.8%

### PheWAS results relating to the genetic risks of artery aneurysms

First, we performed three PheWASs to identify RVFs that were associated with the GRS of AAA, TAA and ICA, respectively. Nine RVFs that represent the vessel calibre (maximum, mean, stander deviation of central or overall vessel calibre), complexity (number of vascular trees) and tortuosity (vessel curvature) were associated with AAA GRS after Bonferroni correction. Of these, ‘ntreeA’, which refers to the number of artery vessels passing through the optic disc, demonstrated the strongest association with AAA GRS (*β* = −0.36, *P* = 6.75e−10) (Fig. 2A). In the sensitivity analysis, after additional adjusting for SBP, smoking, deprivation score and refractive status, four RVFs representing vessel calibres were no longer significant and only five RVFs remained significant (Supplementary Table 4). For TAA and ICA, only the mean artery calibre (‘w_mean_mean_a’) (*β* = 0.05, *P* = 8.37e−05) and the number of vascular trees in the optic disc (‘ntreeA’) (*β* = −0.11, *P* = 5.51e−06) were associated with the genetic risk of thoracic and intracranial aneurysms, respectively. The ‘ntreeA’ was common for both AAA and ICA (Fig. 2C). The association between ‘w_mean_neam_a’ and TAA GRS disappeared after adjusting for more covariates in the sensitivity analysis (Supplementary Table 4).

**Fig. 1:**
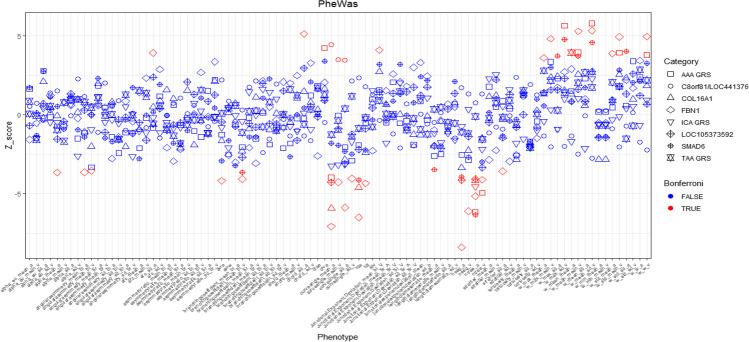
Manhattan plot for the PheWAS of genetic aneurysm risks with RVFs. The x-axis represents the 91 RVFs, y-axis represent the *Z*-score (*Z*-score = *β*/*SE*) of the PheWAS findings. Different symbols represent different genetic risks of aneurysm, the blue/red colour represents whether the *P* value is passing the Bonferroni correction. AAA, abdominal aortic aneurysm; TAA, thoracic aortic aneurysm; ICA, intracranial aneurysm

In summary, in the main analysis, we found that ten unique RVFs were associated with the genetic risk of the three types of aneurysms (Fig. 1). The majority of the RVFs were associated with AAA GRS, and ‘ntreeA’ was associated with both AAA and ICA.

### PheWAS results relating to the genetic risks of Marfan syndrome

**Fig. 2:**
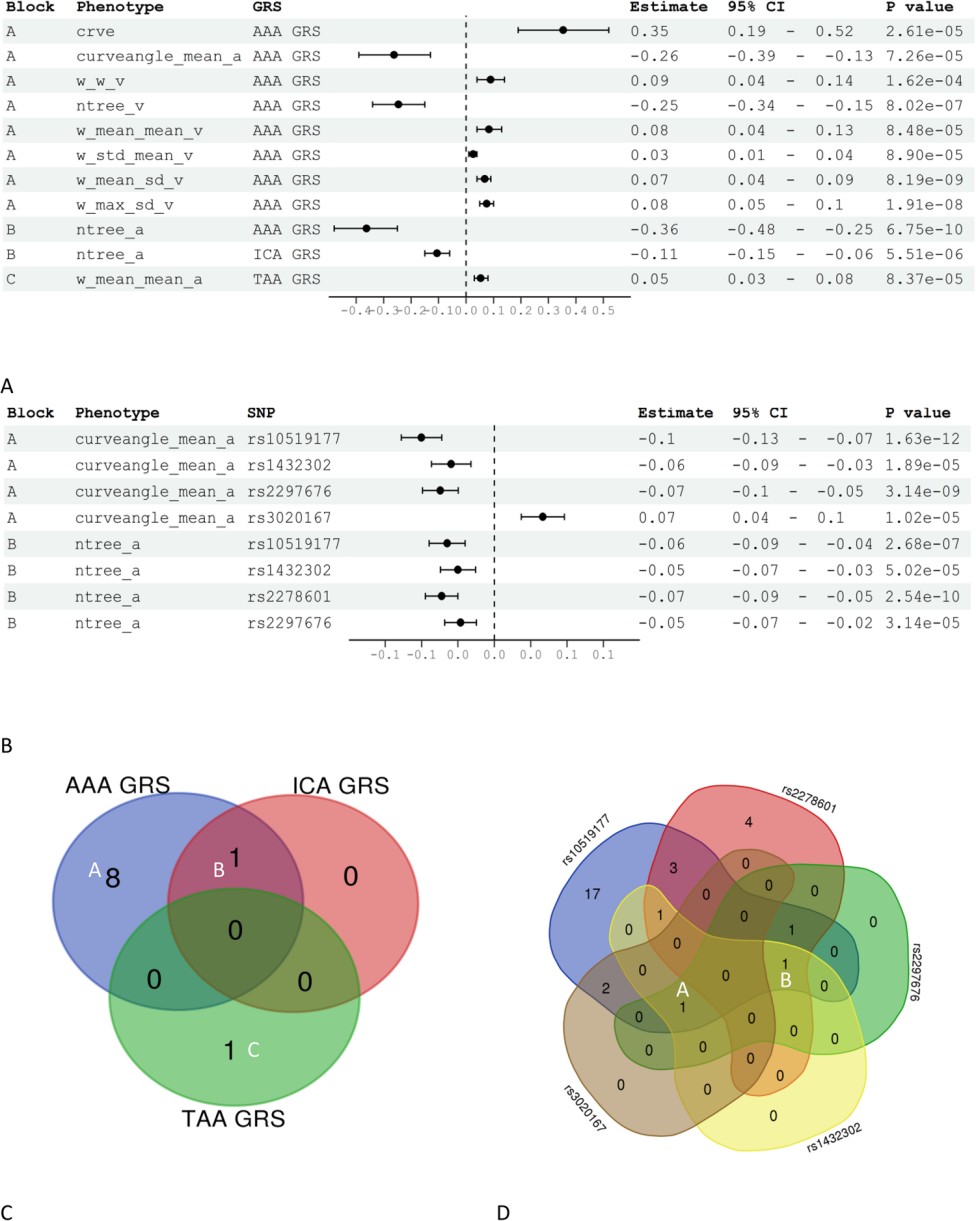
RVFs associated with aneurysm GRSs, and MFS SNPs identified by PheWAS. **A** Forest plot demonstrating the significant RVFs that were identified by PheWAS of AAA/TAA/ICA GRSs; **B** Forest plot demonstrating the most common RVFs that were identified by PheWAS of MFS SNPs; **C** Venn diagram of the PheWAS results showing the common RVFs associated with AAA/TAA/ICA GRSs; ‘A/B/C’ reflects the corresponding blocks of RVFs shown in **A**; **D** Venn diagram of the PheWAS results showing the common RVFs associated with MFS SNPs; ‘A/B’ reflects the corresponding blocks of RVFs shown in **B**

We then performed five PheWASs to identify RVFs that were associated with five different MFS genes, respectively. For the most common MFS gene *FBN1*, 26 RVFs were associated with the mutation of the corresponding SNP, and the number of artery segments (‘nseg_a’, reflecting vessel complexity) showed the strongest association (*β* = −1.67, *P* = 5.07e−17). After adjusting for covariates, especially refractive status, 21 RVFs remained significant (Fig. 2B, D, Supplementary Table 5). For the remaining MFS SNPs, three RVFs for rs2297676 (*COL16A1*), three RVFs for rs1432302 (*LOC105373592*), three RVFs for rs3020167 (*C8orf81/LOC441376*) and ten RVFs for rs2278601 (*SMAD6*) were identified in the main PheWAS analysis (Fig. 1). Sensitivity analysis revealed similar findings (Supplementary Table 5).

For all significant RVFs identified by different MFS genes, there were some overlaps. For example, ‘curveangle_mean_a’, the mean segment angles between each artery branch at a length of ten pixels, was common for four MFS SNPs (*FBN1_*rs10519177: *β* = −0.10, *P* = 1.63e−12; *COL16A1_*rs2297676: *β* = −0.07, *P* = 3.14e−09; *LOC105373592_*rs1432302: *β* = −0.06, *P* = 1.89e−05; *C8orf81/LOC441376_*rs3020167: *β* = 0.07, *P* = 1.02e−05). And ‘ntreeA’ was shared by four other MFS SNPs (*FBN1_*rs10519177: *β* = −0.06, *P* = 2.68e−07; *COL16A1_*rs2297676: *β* = −0.06, *P* = 3.14e−05; *LOC105373592_* rs1432302: *β* = −0.05, *P* = 5.02e−05; *SMAD6*_rs2278601: *β* = −0.07, *P* = 2.54e−10) (Fig. 2B and D).

Taking all the PheWAS results together, in the main analysis, 32 unique RVFs were associated with the genetic risks of aneurysms and MFS (we referred to these RVFs as aneurysm-RVF) (Fig. 1). In the sensitivity analysis, 26 aneurysm-RVFs were identified (Supplementary Table 4–5). Although each aneurysm GRS or MFS gene was associated with some specific aneurysm-RVFs, we still observed many aneurysm-RVFs that were commonly shared by different aneurysm risks (Supplementary Figure 2).

### Derivation of an aneurysm-RVF risk model and comparison with a clinical risk model

In the PheWAS stage, 32 RVFs were considered as aneurysm-RVFs. To investigate whether these RVFs were capable of predicting future aneurysm events, an aneurysm-RVF risk model was developed and compared with a clinical risk model and a baseline model. Details of the three models are listed in Supplementary Method 3.

In the derivation cohort, 54.2% were women, the mean age was 55.2 years, and the Townsend deprivation score was −1.71 (IQR: 4.28). The majority of the participants never smoked (57.1%) and had a slightly elevated BMI (27.2 ± 4.68 kg/m^2^). The summary statistics for clinical risk and the 32 aneurysm-RVFs are shown in Supplementary Table 6 and 7. There were 59 (0.3%) incident cases of aneurysm during the follow-up period. The incidence of aneurysm was 4.06 (95% CI: 3.09–5.24) per 10,000 person-years, 2.16 (95% CI: 1.26–3.15) per 1000 person-years in women and 6.31 (95% CI: 4.55–8.54) in men per 10,000 person-years.

In all three models, older age and male gender were associated with an increased risk of hospital admission due to aneurysm over the follow-up period. In the clinical risk model, both taking blood pressure-lowering medication (HR: 2.78, 95% CI: 1.43–5.39) and a previous history of aneurysm were identified as significant risk factors (HR: 18.75, 95% CI: 9.93–35.41). In the aneurysm-RVF model, after adjustment for age, sex and previous history of aneurysm, both ‘curveangle_mean_v’ (HR: 1.44, 95% CI: 1.08–1.93) and ‘nseg_v’ (HR: 1.73, 95% CI: 1.03–2.90) were associated with an increased risk of aneurysm. Analysis also indicated that ‘curveangle_sd_v’ (HR: 0.68, 95% CI: 0.50–0.93) was associated with a reduction of risk (Supplementary Table 8–10).

**Fig. 3:**
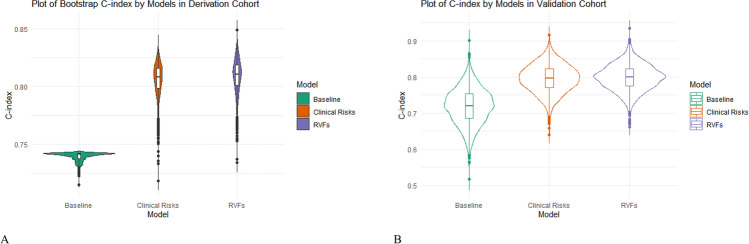
The average *C*-index of the baseline, clinical risks and aneurysm-RVF model from the derivation and validation cohort. **A** Each coloured violin plot represents the average and distribution of *C*-index of different models from the derivation cohort, the *C*-indexes were derived from 2000 bootstrapping: **B** the average and distribution of *C*-index of different models from the validation cohort

After 2000 rounds of bootstrapping within the internal tests, the *C*-index for the baseline model was 0.739 (95% CI: 0.739–0.746); for the clinical risk model, the *C*-index was 0.806 (95% CI: 0.778–0.834) and for the aneurysm-RVF model, the *C*-index was 0.809 (95% CI: 0.780–0.838) (Fig. 3A). In general, in the derivation cohort, the aneurysm-RVF model we developed demonstrated a good performance in predicting the future risk of aneurysm compared to the baseline or clinical risk models.

### Validation of the aneurysm-RVF risk model and comparison with a clinical risk model

The discriminative capability of these models was further examined in a validation cohort. In the validation cohort, there were 28 (0.3%) incident cases; the incidence of aneurysm was 4.55 (95% CI: 3.03–6.58) per 10,000 person-years. The demographic factors, clinical risk factors and aneurysm-RVFs were similar when compared between the derivation and validation cohort (Supplementary Table 6 and 7).

Over 8 years of follow-up, the baseline model yielded a *C*-index of 0.719 (95% CI: 0.620–0.816), the clinical risk model yielded a *C*-index of 0.795 (95% CI: 0.718–0.871), and the aneurysm-RVF model yielded a *C*-index that was similar to the clinical model at 0.798 (95% CI: 0.727–0.869) (Fig. 3B). Although the overall discriminative capabilities were slightly reduced, the aneurysm-RVF model showed equally good performance when compared to the clinical risk model. These results validated the findings in the derivation cohort.

### Predictive capability of aneurysm-RVF score

**Fig. 4:**
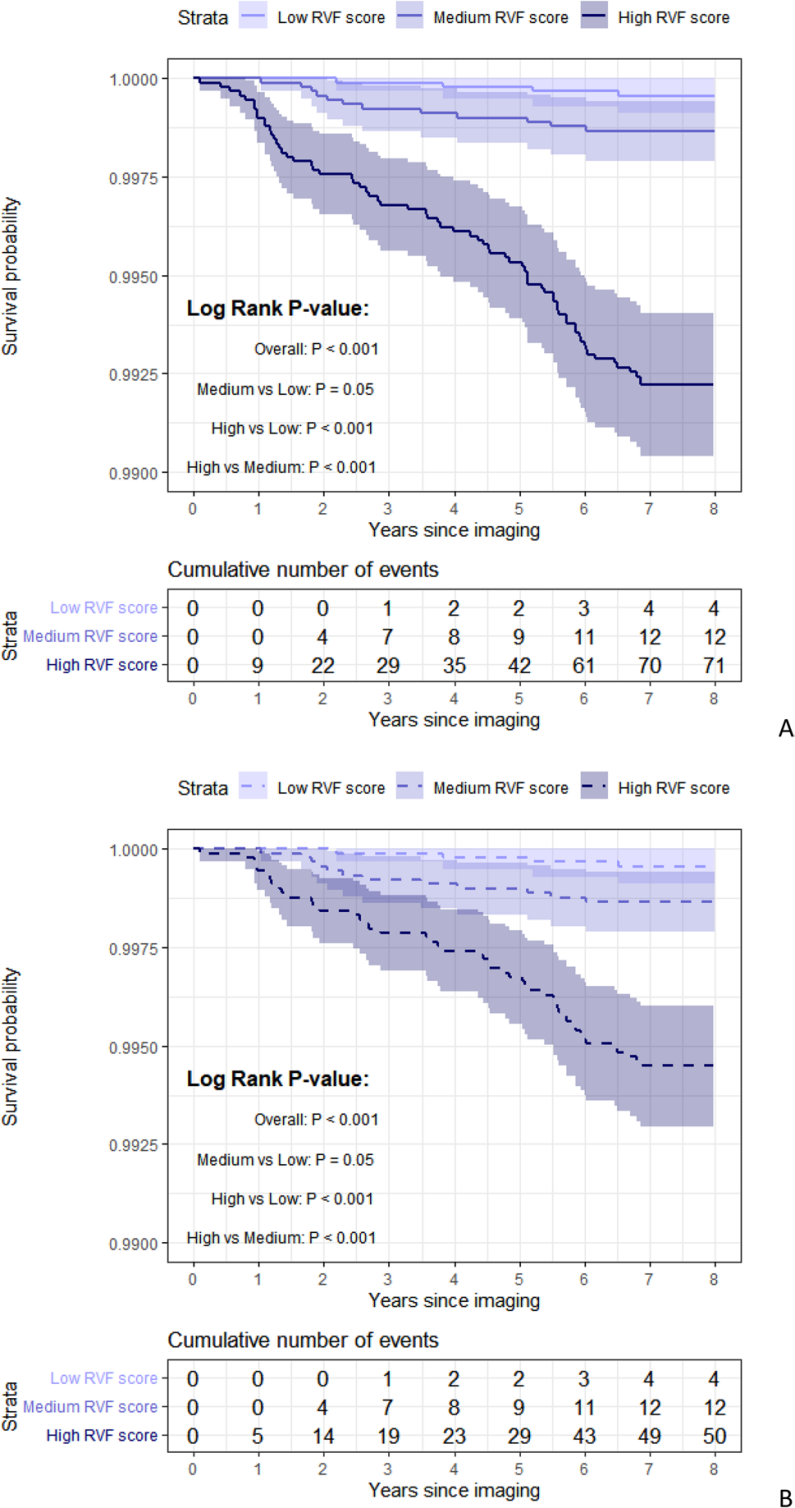
Kaplan-Meier plots for aneurysm risk according to the aneurysm-RVF score. **A** Aneurysm-RVF score in the complete cohort; **B** Aneurysm-RVF score in the participants with first diagnose of the aneurysm

Finally, to investigate the capability of aneurysm-RVFs to identify individuals at risk of aneurysm, an aneurysm-RVF score was predicted for each participant in the complete cohort based on metrics from the derivation cohort. The aneurysm-RVF score was divided into tertiles and a Cox proportional hazard analysis was performed. In the main analysis, compared to the lowest tertile, the estimated hazard ratio was 3.00 (95% CI: 0.97–9.30) for participants in the medium tertile and was 17.80 (95% CI: 6.50–48.75) for participants in the highest tertile. The log-rank test demonstrated that the overall difference in survival rate was statistically significant among the three tertiles (*P* < 0.01); only the difference between the medium and low score groups was boundary significant (*P* = 0.05) (Fig. 4A). In the sensitivity analysis, where participants with previously reported aneurysm were removed, the aneurysm-RVF score yielded slightly smaller estimates: HR was 3.0 (95% CI: 0.97–9.30) for participants in the medium tertile and 12.52 (95% CI: 4.523–34.677) for participants in the highest tertile. The difference was significant among the three tertiles except for comparisons between the medium and low score groups (*P* = 0.05) (Fig. 4B). These findings demonstrated that the aneurysm-RVF score we developed can precisely discriminate subjects at risk of aneurysm.

## Discussion

### Summary of the findings

To our knowledge, this is the first study to innovatively integrate the genetic risk of aneurysm and oculomics of pan-retinal vascular geometry to investigate the value of RVFs in predictive diagnosis of systemic aneurysms. Our PheWAS analysis identified 32 RVFs that were associated with the genetic risks of aneurysms. The identified RVFs were considered to share common biological aetiologies with systemic aneurysms and were used to develop an aneurysm-RVF model to predict the future risk of aneurysm. The aneurysm-RVF model exhibited an equally good performance when compared to a clinical risk model (*C*-index = 0.798 [aneurysm-RVF model] vs. 0.795 [clinical risk model]). The aneurysm risk score derived from our model can successfully stratify subjects at different levels of aneurysm risk (upper vs. lower tertile, HR = 17.80 [6.50–48.75]).

The existing aneurysm screening guidelines only consider a few biomarkers [12, 39] and are generalized for ‘average patients’. In contrast, PPPM aims to improve the outcomes of medical interventions for each individual by developing new medical policies for clear target patients on the basis of population heterogeneity [40]; however, this strategy relies on advances in biomarker discovery [41, 42]. Our study identified new oculomic biomarkers and provided evidence that these biomarkers can be used in the predictive diagnosis of aneurysms. Our strategy can also help to produce a tailored and targeted screening approach to benefit both patients and the healthcare system, thus contributing to the paradigm of PPPM.

### The advantages and rationale of applying an individual aneurysm genetic risk for PPPM

Applying an individual’s genetic information is one of the fundamental steps of the implementation of PPPM although the clinical prospects of this strategy are still being explored. Previously, one of the challenges of applying precision medicine in aneurysm was to translate genomic information to the clinical settings and to develop better diagnostic tools or management setups for aneurysms [43]. The results of our study provided one potential application. There are several advantages of using the genetic risk of aneurysms rather than applying the phenotype itself. Aneurysms are associated with a low prevalence in the general population and are mostly asymptomatic initially. Cases identified from routinely collected phenotypic data would usually underestimate the true prevalence and render the study extremely underpowered. For example, when using phenotypic data, the Atherosclerosis Risk in Communities (ARIC) Study failed to identify any significant association between retinal vasculature and the incidence of AAA [30]. However, in our study, we found that 10 RVFs were associated with the genetic risk of AAA, even after adjusting for additional confounding factors. Of note, the SNPs selected for aneurysms often reveal the biological mechanism of the disease; for example, SNPs selected for AAA encode genes that exert inflammatory and immune function (*IL6R* and *CDKN2BAS1/ANRIL*) or participate in lipoprotein metabolism (SORT1 and LDLR). These processes are important for the development of AAA [44]. The use of aneurysm GRSs would better reflect its biological mechanisms [17]; hence, its association with RVFs would be more biological rather than statistical.

We also note that more RVFs were found to be associated with AAA GRS than TAA. This could be due to the better performance of AAA GRS which included 12 SNPs compared to three or four SNPs included in TAA or ICA GRS. In addition, in terms of embryological origin, abdominal and thoracic aneurysms are different; this causes differing pathologies [45]. The Vascular Smooth Muscle Cells (VSMCs) in the ascending aorta and arch vessels are derived from neural crest stem cells and progenitor cells in the second heart field, while those in the descending aorta are derived from the mesodermal somatic precursor’s layer [46, 47]. It is also known that the retinal vasculature is derived from the mesoderm, which is similar to the descending aorta. Hence, the common embryonic origin between AAA and retinal vessels might also lead to the better performance of AAA GRS.

### Oculomics can reflect systemic disorders

As an emerging research area, oculomics has been applied to predict systemic diseases under the framework of PPPM. The ocular system is enriched with connective (e.g. the sclera), neuron (e.g. the retinal neuron layer) and vascular (e.g. the retinal vessel and choroid) tissues; hence, oculomics can reflect systemic disorders from a variety of aspects. One recent study conducted by Evsevieva et al. demonstrated that connective tissue dysfunction can be manifested as disorders in both systemic vascular system as well as ocular system and cause diseases such as myopia and glaucoma [48]. Similarly, MFS is an autosomal dominant genetic disorder of the connective tissue. In a manner that differs from polygenic diseases in which each SNP only exerts a small effect on the outcome [22, 23], the mutation of MFS genes would cause relatively severe dysfunction of the connective tissues. The corresponding clinical manifestations include arterial aneurysms and high myopia: this could be a potential application of using oculomics of high myopia to predict systemic disease [49]. Furthermore, connective tissue disorder can influence retinal vascular geometry [50]: we suspect that this would lead to another potential application of using oculomics of RVFs to predict aneurysms. In a previous case report, an MFS patient with an *FBN1* mutation developed an aneurysm and was detected with abnormal retinal vascular morphology in both eyes [51]. Another study investigated the retinal vasculature alterations in genetically confirmed MFS and found that the severity of MFS was significantly correlated with impairments in the retinal vasculature [52]. Consistent with these findings, in our study, we observed that (1) the genes with clear biological effects on connective tissue disorders like MFS were also associated with oculomics of RVFs (Supplementary Figure 2); (2) the identified oculomics of RVFs had a capability in predicting systemic aneurysm, which was more precise than baseline information. Our study provides more evidence to support the application of oculomics in systemic disease interventions under the PPPM framework.

One of the concerns is that MFS patients also suffer from high myopia [49]; this might influence the morphology of the retinal vessels and induce a spurious association between RVF and MFS [33]. Hence, in this study, apart from clinical risk factors, refractive error was also carefully adjusted in the model to reduce potential bias caused by myopic status.

### Biological interpretation of aneurysm RVF

One of our key findings is that ‘curveangle_mean’, a parameter that reflects the tortuosity of the retinal vessels, was commonly identified by different aneurysms and MFSs, thus indicating its importance for aneurysm. Previous studies have described variable associations between retinal vessel tortuosity and cardiovascular diseases, albeit with conflicting evidence on occasion. For example, it was reported that increasing retinal arteriolar tortuosity was associated with an increased risk of stroke in type 2 diabetic patients [53] and might also be associated with hypertension and hyperlipidaemia [54]. Sasongko et al. [55] found no association between retinal vessel tortuosity and a range of clinical risk factors, including blood lipids in diabetic patients. However, Cheung and Taarnhøj reported that flatter retinal vessels (smaller in tortuosity) were associated with older age, higher blood pressure as well as higher BMI and these are all risk factors for aneurysm [56, 57]. Similar to Cheung and Taarnhøj’s findings, we found that smaller values of ‘curveangle_mean’ (flatter vessels) were associated with an increased risk of aneurysm. In previous studies, general tortuosity was calculated as the arc-chord ratio of a vessel; this measures a wider range of the vessel arch and is insensitive to the frequency with which a vessel ‘wiggles’ [58]. To overcome this problem, ‘curveangle’ focuses on smaller regions of the vessels by measuring vessel branches sampled at a length of 10 pixels [33]. For simplicity, tortuosity and ‘curveangle’ were standardized to the same direction: the greater the value, the curvier the vessel. We suspect that compared to tortuosity, the ‘curveangle’ might be more sensitive in reflecting the early degeneration of the retinal vessels. However, to what extent this feature can be used for detecting systemic aneurysms needs to be further investigated.

Another interesting finding is that ‘ntreeA’ was negatively associated with arterial aneurysms. This may be relevant to its geometric capability of reducing the blood flow shear stress. Common features of arterial aneurysms include the dysfunction and loss of vascular smooth muscle cells, extracellular matrix degradation and inflammation [59], which can disrupt arterial wall structural integrity, weaken vessels, remodel the arterial wall and subsequent dilate the artery [60]. A high wall shear stress caused by abnormal flow conditions can activate pro-inflammatory signalling in endothelial cells and disrupt the internal elastic lamina and collagen matrix, thereby leading to a focal bulge of the wall and the initiation of arterial aneurysm. It is possible that the increased number of vascular trees may play an important role in diverting and therefore decreasing the shear stress; however, further hemodynamic studies are needed.

### Strength and limitations

The main strength of the present study is that this large and prospective cohort features extensive phenotypic, genotypic and oculomics detail about its participants. In addition, this study used genetic information relating to aneurysms to identify new diagnostic biomarkers that better represented the population; furthermore, these biomarkers were highly robust. We also used a deep learning system to analyse large quantities of retinal images which can automatically measure a wide range of RVF parameters. However, the findings of this study need to be interpreted with caution due to the following of the following limitations. Firstly, participants involved in the UK Biobank study might not be fully representative because extremely poor-health individuals could not participate in this study. Secondly, the gene panel for MFS included a myopia gene; although the refractive error of the eye was adjusted, the pleiotropic effect of the genes can still lead to potential bias. Finally, since patients that were diagnosed with aneurysms in the UK Biobank data set were rare, and participants who had retinal images were younger on average, replication in other studies will give better clinical indications of these findings.

## Conclusion and expert recommendations for the framework of PPPM

Our study showed that RVFs, quantified based on retinal images, were associated with the genetic risks of aneurysms, and that the aneurysm-RVF score can efficiently identify patients with risks of aneurysms. Our findings support the further development and application of PPPM in the medical intervention of aneurysms from different perspectives. Firstly, from the aspect of disease prediction, conventional prediction models mainly rely on clinical risk factors while our study innovatively implemented oculomics and identified non-invasive biomarkers for aneurysm prediction, thus enabling the construction of more practical prediction models. Secondly, from the aspect of primary aneurysm prevention, the current screening strategies for AAA are mainly based on age and sex, while for other types of aneurysms, effective screening strategies are still required. The aneurysm-RVF score can effectively stratify patients at risk. Furthermore, these imaging biomarkers are safe and inexpensive, thus helping us to monitor the progression of aneurysms in a long-term and dynamic manner. Thirdly, from the aspect of personalization, as the oculomics of RVF is easily accessible compared to other medical examinations, applying oculomics for the detection of aneurysm would benefit the majority of the population, especially for younger patients or those living in areas with limited medical resources.

Finally, although the aneurysm-RVFs identified from our study demonstrated significant potential as reliable biomarkers, their biological causes remain unclear. Further studies are now warranted to confirm the clinical value of RVFs in the screening and early diagnosis of arterial aneurysms.

## Supplementary information

ESM 1

ESM 2

## Abbreviations

*AAA*: Abdominal Aortic Aneurysm
*TAA*: Thoracic Aortic Aneurysm
*ICA*: Intracranial Aneurysm
*MFS*: Marfan Syndrome
*RVF*: Retinal Vascular Feature
*PheWAS*: Phenome-Wide Association Analyses (s)
*GRS*: Genetic Risk Score
*SNP*: Single Nucleotide Polymorphism
