# Supplementary material for: Integrating oculomics with genomics reveals imaging biomarkers for preventive and personalized prediction of arterial aneurysms
Source: EPMA J. 2023 Feb 13;14(1):73–86. doi: 10.1007/s13167-023-00315-7 (PMC9971392; doi:10.1007/s13167-023-00315-7)
Supplement: Supplementary file 1 — ESM 1 [file 13167_2023_315_MOESM1_ESM.docx]

**Integrating genetics with oculomics reveals imaging biomarkers for personalized arterial aneurysm risk prediction**

**Supplementary Method 1: RVF extraction**

For each retinal image, Retinal-based Microvascular Health Assessment System (RMHAS) was used to extract the features. First, the image quality was classified into three grades (good, usable and reject) using a convolutional neural network. The images with a grade of “reject” were excluded. Second, the retinal artery, vein and optic disc were segmented by the U-Net component from W-Net. Images were further excluded if they were without detectable optic disc, with less than 6 arteries or veins detectable in a zone 0.5-1.0-disc diameter from the margin of the optic disk, or less than 2 arteries or veins in the whole fundus. Last, the retinal vascular features were measured by using custom region-specific summarization and global/geometric parameters, including the calibre, complexity, length, tortuosity and branching angle of the retinal artery and vein ^[1]^.

**Supplementary Method 2: Characteristics associated with aneurysm risk**

At the baseline, trained nurses measured SBP twice using a digital sphygmomanometer (Omron 705 IT; OMRON Healthcare Europe B.V., Hoofddorp, Netherlands), and the average of the two measurements was used in the analysis. DBP was then categorized as above 90mmHg or below. Body weight was measured by a Tanita BC-418MA body composition analyser (Tanita Corporation, Arlington Heights, IL) and body height was measured in a barefoot standing position using the Saca 202 device. BMI was calculated accordingly by dividing the body weight (kilograms) by height squared (meters). Demographic information such as age at baseline, gender, Townsend deprivation score and smoking status were collected by a touchscreen computer. Medications were derived from verbal interview in the assessment center. Smoking status was categorized as never, former, or current smoker.

**Supplementary Method 3: Models used for prediction.**

Models were build on R coding

- **Baseline Model** <- formula(Surv(t.fu, i.fu) ~ age + sex);
- **Clinical Risk Model** <- formula(Surv(t.fu, i.fu) ~ age + sex + bmi + townsend + sbp + dbp + hba1c + tc + hdl + ldl + trig + i.drg.bp + i.drg.dm + i.drg.lpd + c.smok + cvd + i.pre + hbp + diabetes+ lpd);
- **Aneurysm-RVF Model** <- formula(Surv(t.fu, i.fu) ~ age + sex + i.pre + alpha_mean_a+ angularasymmetry_mean_bi_a + angularasymmetry_mean_bi_v + arc_sd_a + avre + branchingcoefficient_mean_bi_v + chord_sd_a + crve + curveangle_mean_a + curveangle_mean_v + curveangle_sd_a + curveangle_sd_v + fda + fdt + lengthdiameterratio_mean_a + nseg_a + nseg_v + ntree_a + ntree_v + strahler_sd_a + Junctional.Exponent.Deviation_mean_bi_V + w_max_mean_a + w_max_mean_v + w_max_sd_a + w_max_sd_v + w_mean_mean_a + w_mean_mean_v + w_mean_sd_v + w_std_mean_a + w_std_mean_v + w_std_sd_a + w_w_v ).

Where:

Baseline information:

- t.fu: time to follow up;
- i.fu: event of aneurysm, yes=1,no=0;
- Age: age at recruitment;
- Sex: female = 0, male = 1;

Contineuous variables:

- bmi: baseline BMI;
- townsend: Townsend score
- dbp: systolic blood pressure, average value of the baseline measurement;
- dbp:systolic blood pressure, average value of the baseline measurement;
- hba1c: glycated hemoglobin, in mmol/mol;
- tc: blood lipid measuments of total cholesterol, in mmol/L;
- hdl:blood lipid measuments of high density cholesterol, in mmol/L;
- ldl:blood lipid measuments of low density cholesterol, in mmol/L;
- trig:blood lipid measuments of triglycerides, in mmol/L;

Category variables:

- i.drg.bp: use of any blood pressure lowering medication, binary, 0=no, 1=yes;
- i.drg.dm: use of any anti diabetic medication, binary, 0=no, 1=yes;
- i.drg.lpd: use of any blood lipid lowering medication, binary, 0=no, 1=yes;
- c.smok: smoking status 0=nonsmoker, 1=ex-smoker, 2=current smoker;
- cvd:previous history of CVD, binary, 0=no, 1=yes;
- i.pre:previous history of aneurysm, binary, 0=no, 1=yes;
- hbp:previous history of hypertension, binary, 0=no, 1=yes;
- diabetes:previous history of diabetes,type 1 or type 2, binary, 0=no, 1=yes;
- lpd:previous history of hyperlipidemia, binary, 0=no, 1=yes;

RVFs: details of the explanation of the RVFs are in supplementary table 1.

**Supplementary Figures**


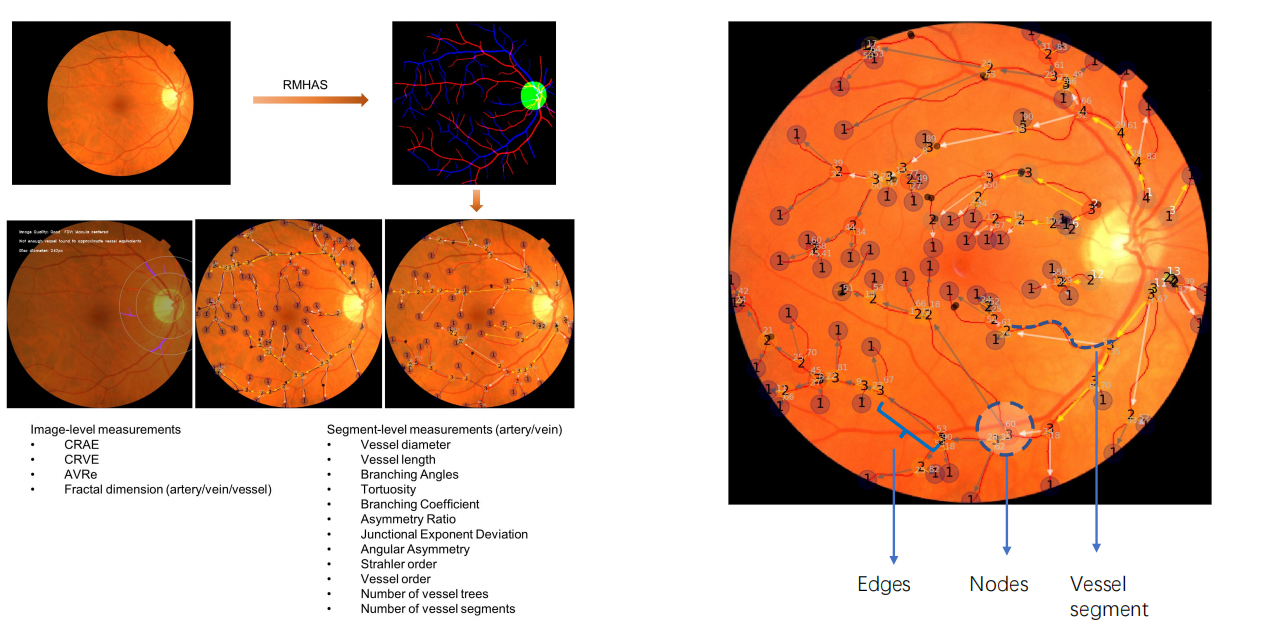


**Supplementary Figure 1 : Analyzing pipeline of the RMHAS.** RMHAS accesses the quality of a retinal image, then 1) cropped to the field of view and resized to 512 ×512 pixels; 2) perform a optic disc and vessel segmentation; 3) measured retinal vessel morphology, which include a) image-level measurements: the vessel calibers summarized as central retinal artery equivalent (CRAE) and central retinal vein equivalent (CRVE); Artery to vein ratio from equivalents (AVRe), and vessel fractal dimension; b) Segment-level measurements (vessels segments separated by the branchings or nodes): the diameters (mean, standard deviation [SD]), arc length, chord length, length diameter ratio (LDR), tortuosity, branching angle (BA), branching angle from edges (BA_edge), branching coefficient (BC), angular asymmetry (AA), asymmetry ratio (AR), junctional exponent deviation (JED) ^[1]^.

**FBN1: 14 RVFs**

nseg_v, curveangle_sd_v, avre, angularasymmetry_mean_bi_v, angularasymmetry_mean_bi_a, w_max_mean_v, Junctional.Exponent.Deviation_mean_bi_V, w_max_mean_a, arc_sd_a, fdt, alpha_mean_a, chord_sd_a, strahler_sd_a,w_std_mean_a

**AAA_GRS & FBN1: 3 RVFs**

ntree_v, w_w_v, w_std_mean_v

**FBN1 & Rest MFS genes: 5 RVFs**

Fda, branchingcoefficient_mean_bi_v, curveangle_mean_v, nseg_a, curveangle_sd_a

**Rest MFS genes: 3 RVFs**

w_std_sd_a, lengthdiameterratio_mean_a,w_max_sd_a

**AAA_GRS: 1 RVFs**

crve

**TAA_GRS: 1 RVFs**

w_mean_mean_a

**AAA_GRS & Rest MFS genes : 1 RVFs**

w_max_sd_v

**AAA_GRS & Rest MFS genes & FBN1 : 3 RVFs**

curveangle_mean_a, w_mean_mean_v,

w_mean_sd_v

**AAA_GRS & Rest MFS genes & FBN1 & ICA_GRS : 1 RVFs**

ntree_a


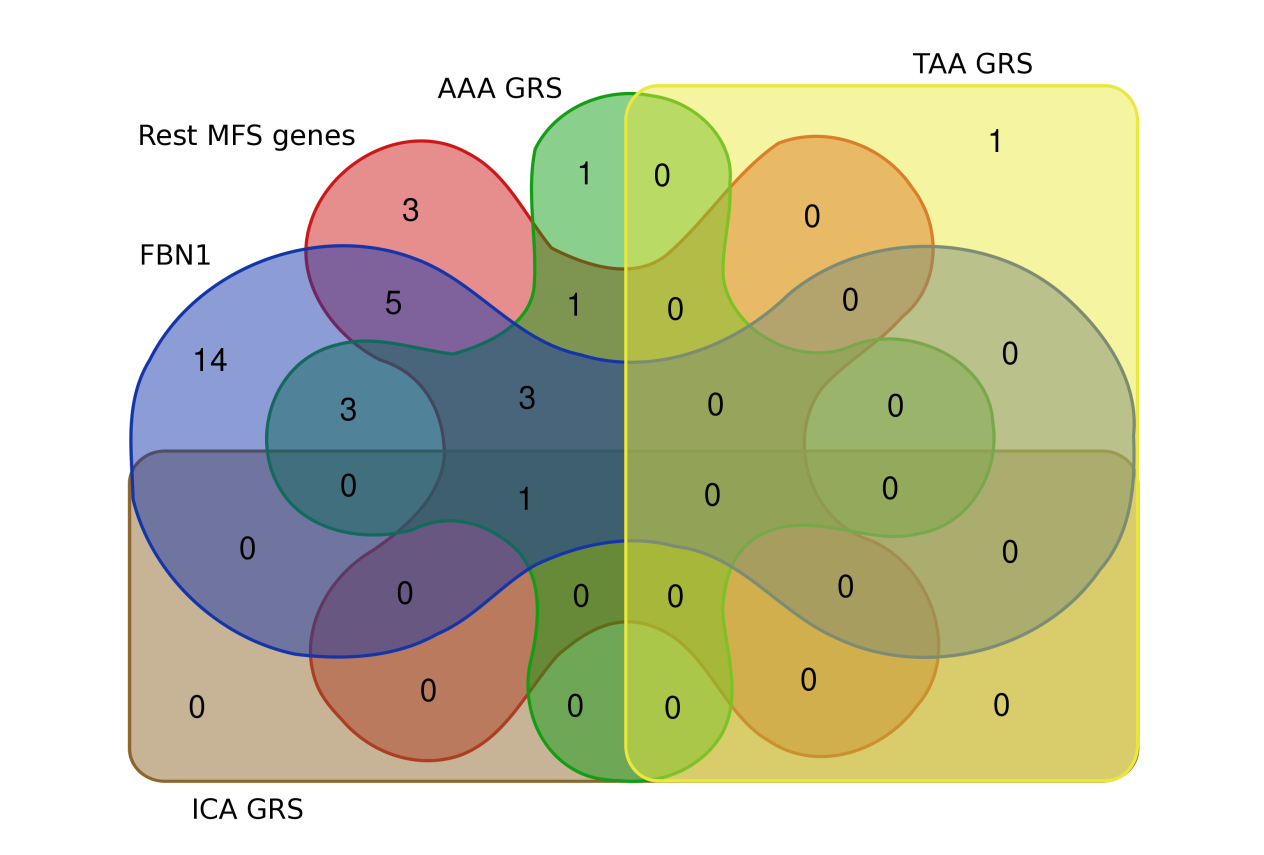


**Supplementary Figure 2: Venn diagram of the overall PheWAS results.** Venn diagram showing the common RVFs associated with AAA/TAA/ICA GRSs and MFS genes.

Reference:

[1] Shi D, Lin Z, Wang W, et al. A Deep Learning System for Fully Automated Retinal Vessel Measurement in High Throughput Image Analysis. *Front Cardiovasc Med* 2022; **9**: 823436.
